# Economic Evaluations of Adult Male Circumcision for Prevention of Heterosexual Acquisition of HIV in Men in Sub-Saharan Africa: A Systematic Review

**DOI:** 10.1371/journal.pone.0009628

**Published:** 2010-03-10

**Authors:** Olalekan A. Uthman, Taiwo Aderemi Popoola, Mubashir M. B. Uthman, Olatunde Aremu

**Affiliations:** 1 The West Midlands Health Technology Assessment Collaboration (WMHTAC), Department of Public Health, Epidemiology & Biostatistics, University of Birmingham, Birmingham, United Kingdom; 2 Research and Evaluation, Center for Evidence-Based Global Health, Birmingham, United Kingdom; 3 Department of Community Health and Epidemiology, University of Ilorin, Ilorin, Kwara State, Nigeria; 4 Department of Public Health Sciences, Division of Social Medicine, Karolinska Institutet, Stockholm, Sweden; Erasmus University Rotterdam, Netherlands

## Abstract

**Background:**

There is conclusive evidence from observational data and three randomized controlled trials that circumcised men have a significantly lower risk of becoming infected with the human immunodeficiency virus (HIV). The aim of this study was to systematically review economic evaluations on adult male circumcision (AMC) for prevention of heterosexual acquisition of HIV in men.

**Methods and Findings:**

Studies were identified from the following bibliographic databases: MEDLINE (Ovid), EMBASE (Ovid), Cochrane Library (Wiley's internet version), NHS EED and DARE Office of Health Economics HEED. The searches were conducted in November 2009. The Drummond 10-point checklist was used for methodological critique of the economic evaluations. Cost data were inflated and converted to 2008 US dollars (US$). Of 264 identified papers, only five met the inclusion criteria and were included in the review. The studies were published between 2006 and 2009. Most of the studies were carried out from the perspective of government healthcare payer. The time horizon ranged from 10 to 20 years. All studies reported that AMC is cost-effective. The reported cost per HIV infection averted ranged from US$174 to US$2808. The key driver of the cost-effectiveness models was circumcision efficacy.

**Conclusions:**

All published economic evaluations offered the same conclusion that AMC is cost-effective and potentially cost-saving for prevention of heterosexual acquisition of HIV in men. On these grounds, AMC may be seen as a promising new form of strategy for prevention of HIV and should be implemented in conjunction with other evidence-based prevention methods.

## Introduction

Male circumcision is one of the oldest and most common surgical procedures worldwide. It may be undertaken for religious, cultural, social and medical reasons. There is conclusive evidence from observational data [1], [2], [3], [4], [5], [6] and three randomized controlled trials [7], [8], [9] that circumcised men have a significantly lower risk of becoming infected with the human immunodeficiency virus (HIV). Several reviews have been reported on the effectiveness of adult male circumcision (AMC) for prevention of heterosexual acquisition of HIV in men [10], [11], [12], [13], [14], [15], [16], [17], [18], [19], [20]. While the literature has focused on the effectiveness alone, we argue that there has not been commensurate interest in economic evaluations. Economic evaluation provides a useful framework to assist policy makers in allocating resources across competing needs. HIV/AIDS is a considerable burden on society resources, and prevention provides a cost-beneficial solution to address these consequences [21]. Sub-Saharan Africa is more heavily affected by Human immunodeficiency virus (HIV) and acquired immunodeficiency syndrome (AIDS) than any other region of the world [22]. Estimated 22.5 million people were living with HIV at the end of 2007 and approximately 1.7 million additional people were infected with HIV during that year [22]. In just the past year, the AIDS epidemic in Africa had claimed the lives of estimated 1.6 million people in this region. More than 11 million children have been orphaned by AIDS [22].

To our knowledge, no systematic review of the peer-reviewed economic evaluations literature on AMC for prevention of heterosexual acquisition of HIV in men has been published to date. A goal of systematic review is to provide the clinician, researcher or policy makers with a balanced appraisal of the totality of the evidence in an area by reading one review, contrary to a multitude of individual studies. This allows for a more objective appraisal of the evidence, which may lead to resolution of uncertainty and disagreement. Therefore, the objectives of this systematic review were to identify published economic evaluations on AMC for prevention of heterosexual acquisition of HIV in men and to identify areas that merit further study.

## Methods

### Eligibility Criteria

Inclusion and exclusion criteria applied for economic searches are summarised below:

- **Study design**: cost-effectiveness analysis, cost-utility analysis, or cost-benefit analysis
- **Population**: adult male in sub-Saharan Africa
- **Intervention**: adult male circumcision (AMC)
- **Comparator**: no AMC

### Information Sources and Search strategy

A comprehensive search for literature on the cost-effectiveness of AMC versus no AMC for prevention of heterosexual acquisition of HIV in men was conducted. Studies were identified from the following bibliographic databases: MEDLINE (Ovid), EMBASE (Ovid), Cochrane Library (Wiley's internet version), NHS EED and DARE Office of Health Economics HEED. The searches were conducted in November 2009. Searches were not limited by date and there were no language restrictions. Search keywords included male circumcision, circumcision, and adult male circumcision. These keywords were combined with an economic study filter based on the Centre for Review Disseminations model [23], [24]. We also checked the reference lists of all studies identified by the above methods.

### Study Selection

One reviewer applied the inclusion and exclusion criteria. These were checked by a second reviewer. Disagreements were resolved by discussion.

### Data Collection Process and Data Items

For each identified study that met the selection criteria, the following data were extracted country, study design, population, intervention, comparator, perspective, model type, primary outcome, discount rate, time horizon, and price year on to an Excel spreadsheet. The main characteristics, quality assessment (see below) and results of included economic evaluations were tabulated. One review author extracted data and the second author checked the extracted data.

### Quality Assessment

Identified economic evaluations were also assessed against the Drummond 10-point checklist [25]. The checklist was developed to assess/critique the quality of an economic evaluation. Drummond checklists consider the following: description of interventions; study design; identification, measurement and valuation of costs and consequences; discounting; a clear results with sensitivity and uncertainty analysis; and discussion of results in the context of policy relevance and existing literature.

### Summary Measures

For the primary outcome, the preferred measures were cost per HIV infection averted (HIA), and incremental cost-effectiveness ratio (cost per quality adjusted life years (QALY) gained and cost per disability adjusted life years (DALY) averted). Net cost saving was also considered.

In order to make different cost data comparable, the cost data were inflated to 2008 prices using the prices inflation index [26]. For those studies that did not report price year, the year the study was published was used for inflation of cost data. This review was performed according to the Preferred Reporting Items for Systematic Reviews and Meta-Analyses (PRISMA) [27], [28].

## Results

### Study Selection

**Figure 1:**
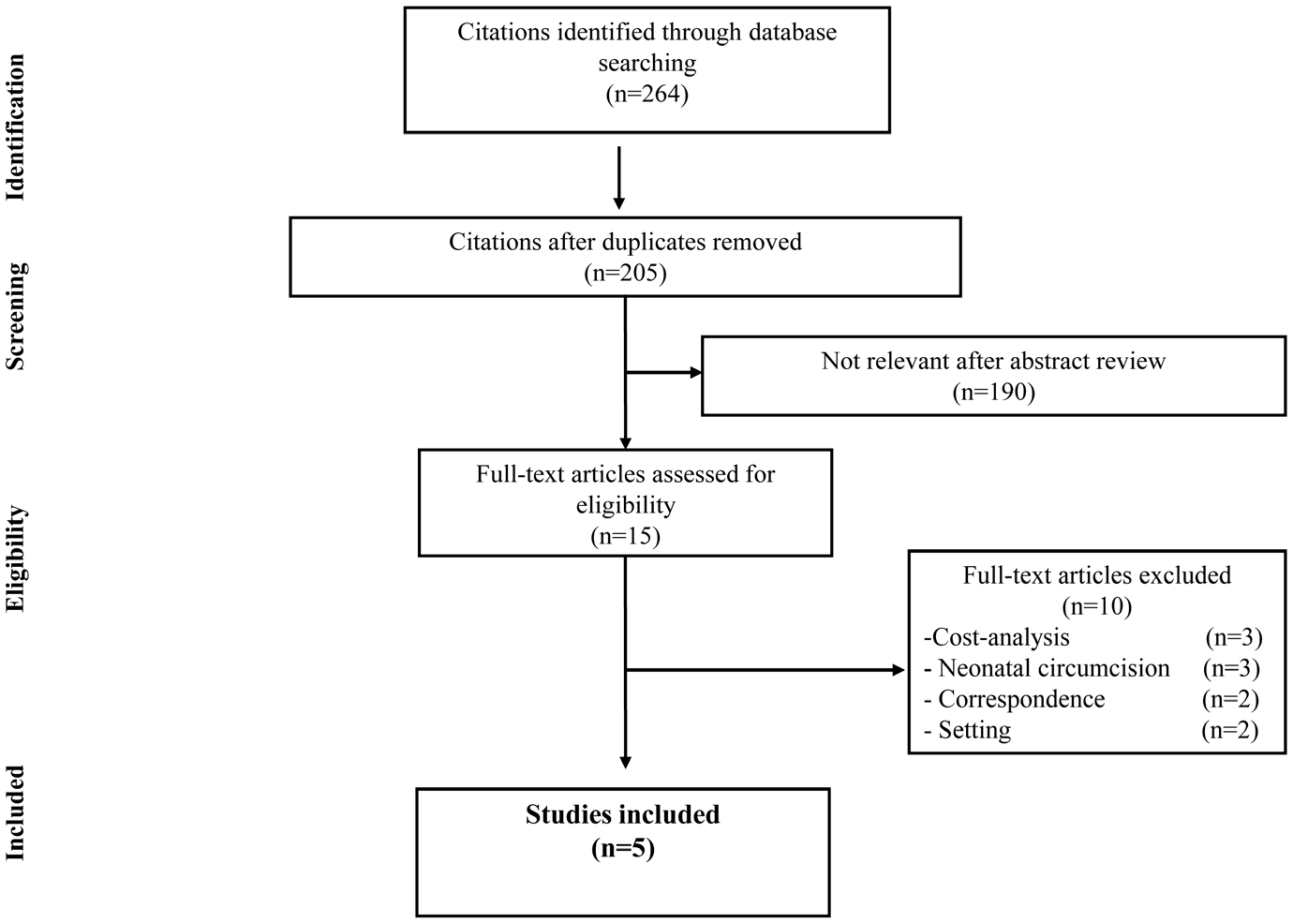
Flowchart for study selection.

The search of electronic databases produced a total of 264 citations (Figure 1). After adjusting for duplicates 205 remained. Of these, 190 studies were discarded because after reviewing the abstracts it appeared that these papers clearly did not meet the inclusion criteria. The full text of the remaining 15 citations was examined in more detail. Ten studies were discarded for the following reasons: cost-analysis (n = 3) [29], [30], [31], neonatal circumcision (n = 3) [32], [33], [34], correspondence (n = 2) [35], [36], and population based on high-income country (n = 2) [37], [38]. Five studies [39], [40], [41], [42], [43] met the inclusion criteria and were included in the systematic review.

### Study Characteristics

Table 1 shows the characteristics of the included studies. The studies were published between 2006 and 2009. Studies population was from Uganda [40], South Africa [39], Mozambique [42], and Botswana [43]. Auvert et al 2008 [41] included data from the following 16 countries in sub-Saharan Africa: Botswana, Burundi, Central African Republic, Kenya's Nyanza province, Lesotho, Liberia, Malawi, Mozambique, Namibia, Rwanda, South Africa, Swaziland, Tanzania, Uganda, Zambia and Zimbabwe. All studies [39], [40], [41], [42], [43] were cost-effectiveness analyses and primary outcome was cost per HIV infection averted (HIA). Most studies were carried out from the perspective of government healthcare payer. All studies [39], [40], [41], [42], [43] compared cost-effectiveness of adult male circumcision (AMC) versus no AMC. The model types included epidemiologic [39], [41], [43], stochastic [40], and costing model [42]. Four studies used 3% discount rate [39], [41], [42], [43]. One study did not report discount rate [40]. The time horizon ranged from 10 to 20 years.

**Table 1:** General characteristics of included studies.

	Study ID				
Characteristics	Gray 2007 [40]	Kahn 2006 [39]	Auvert 2008 [41]	Fieno 2008 [42]	Bollinger 2009 [43]
**Country**	Uganda	South Africa	SSA	Mozambique	Botswana
**Study design**	CEA	CEA	CEA	CEA	CEA
**Intervention**	AMC	AMC	AMC	AMC	AMC
**Comparator**	No AMC	No AMC	No AMC	No AMC	No AMC
**Perspective**	Not reported	government healthcare payer	government healthcare payer	government healthcare payer	government healthcare payer
**Model**	Stochastic simulation	Epidemiologic model	Epidemiologic model	Costing model	Epidemiologic model
**Primary outcome**	Cost per HIA	Cost per HIA	Cost per HIA	Cost per HIA	Cost per HIA
**Discount rate (%)**	NR	3	3		3
**Time horizon (years)**	10	20	10–20	20	17
**Results**					
**Cost per HIA(US$)** *	2808	193	174	390	642

HIA: HIV infection averted; CEA: Cost-effectiveness Analysis; AMC: Adult male circumcision; SSA: sub-Saharan Africa.

*Value in 2008 US dollars.

### Validity Assessment

Details of the quality of assessments can be found in Table 2.

**Table 2:** Quality of included studies.

	Study ID				
Criteria	Gray 2007 [40]	Kahn 2006 [39]	Auvert 2008 [41]	Fieno 2008 [42]	Bollinger 2009 [43]
**1) Well defined question stated?**	yes	Yes	Yes	Partially	Partially
**2) Description of alternative given?**	Yes	Yes	Yes	Yes	Yes
**3) Evidence of effectiveness established?**	Cannot tell	Yes	Yes	Yes	Yes
**4) Relevant costs and outcomes identified?**	Partially	Partially	Partially	Partially	Partially
**5a) Costs measured accurately?**	No	Yes	Yes	Yes	Yes
**5b) Outcomes measured accurately?**	Yes	Yes	Yes	Yes	Yes
**6a) Costs valued credibly?**	Yes	Yes	Yes	Yes	Yes
**6b) Outcomes valued credibly?**	Yes	Yes	Yes	Yes	Yes
**7a) Costs discounted?**	No	Yes	Yes	no	Yes
**7b) Outcomes discounted?**	No	Yes	Cannot tell	no	No
**8) Incremental analysis performed**	Yes	Yes	Yes	Yes	Yes
**9) Sensitivity analysis performed**	Yes	Yes	Yes	No	Yes
**10) Results presented clearly and discussed?**	Yes	Yes	Yes	yes	Yes

#### 1. Was a well-defined question posed in answerable form?

Three studies [39], [40], [41] had clearly defined questions, i.e., to assess the cost-effectiveness of AMC in prevention of HIV. Two studies did not state their research question clearly [42] [43].

#### 2. Was a comprehensive description of the competing alternatives given?

All studies [39], [40], [41], [42], [43] described competing alternatives, AMC versus no AMC.

#### 3. Was the effectiveness of the programmes or services established?

Evidence of effectiveness came from the observational studies or from the trial results [7], [8], [9]. Kahn et al 2006 [39] defined effectiveness as the number of HIA, which was estimated by dynamically projecting over 20 years the reduction in HIV incidence observed in the Orange Farm [7](OF, Gauteng Province, South Africa). Auvert et al 2008 [41] and Fieno et al 2008 [42] also used evidence of effectiveness from OF trial [7]. Bollinger et al 2009 [43] used evidence from three trials [7], [8], [9]. Gray et al 2007 [40] effectiveness parameters were based on an observational study in a rural population of Rakai District, south-western Uganda. Gray et al 2007 [40] assumed that circumcision might reduce the incidence of HIV incidence rate ratios (IRR) varying from 0.3 to 0.6. Efficacy, the reduced incidence of HIV afforded by circumcision, was estimated from 1-IRR.

#### 4. Were all the important and relevant costs and consequences for each alternative identified?

All studies [39], [40], [41], [42], [43] considered cost of AMC. Except for Gray et al 2007 [40], studies [39], [41], [42], [43] considered lifetime medical cost of treating HIV. Only one study included complications associated with AMC in the cost-effectiveness model [39].

#### 5. Were costs and consequences measured accurately in appropriate physical units?

Most studies estimated cost of AMC and lifetime medical treatment of HIV from published studies. Number of HIV infections averted was the primary outcome in all studies. Fieno et al 2008 [42] also valued outcome in DALYs averted. Most studies extrapolated the number of HIV infection averted beyond the trials period.

#### 6. Were costs and consequences valued credibly?

Kahn et al. 2006 [39] cost of performing an AMC was based on the estimate from the OF RCT [7]. Kahn et al. 2006 [39] assumed zero additional training and physical infrastructure development costs in connection with high levels of circumcision coverage. The cost per AMC was varied by ±50% to reflect these potential efficiencies or inefficiencies of scale-up. The cost of adverse events was included and standardized for 1,000 individuals. The lifetime cost of HIV treatment was based on Cleary et al. [44] data from pilot clinics (in South Africa) for a prospective disease state model. Cleary and colleagues [44] estimated a lifetime discounted cost of US$11,948 with antiretroviral therapy (ART) and US$3,793 without ART. However, Kahn et al. 2006 [39] used US$8,000 as the base case value, implying 50% access to ongoing ART, and explored a wide range from US $4,000 to US$12,000.

Gray et al 2007 [40] used the estimated cost per surgery in the Rakai trial [45] (US$69) to estimate the cost per HIA over a period of 10 years. Auvert et al 2008 [41] explored a public cost scenario assuming the use of government health infrastructures only and a private cost scenario assuming reliance on private health care providers only. Program costs were composed of initial and annual costs. In the public cost scenario, initial costs were per circumcision facility (for medical equipment and certification) and for training circumcisers. Annual costs included the oversight and promotion of AMC, the salaries of full-time circumcisers, surgical staff and counsellors, the direct non-salary cost of each AMC (i.e., surgical supplies), facility overhead (i.e., operating costs) and program overhead. In the private cost scenario, all facility-level costs were included in the price per AMC paid to providers. No initial costs were included because these providers were already equipped to perform AMC. The price of each AMC covered salaries of circumcisers, other health staff, counselling, surgical supplies, follow-up, treatment of adverse events and operating costs. In addition to direct provider payments, Auvert et al 2008 [41] also assumed annual program overhead costs of 10% to cover the public promotion of AMC. In order to estimate costs and savings from HIV treatment, Auvert et al 2008 [41] assumed that 30% of HIV-infected individuals eligible for antiretroviral treatment were receiving it. The averted cost of medical treatment for HIV over time was a function of the number of HIV infections averted each year and the rate of disease progression, combined with associated medical costs.

Fieno et al 2008 [41] reported detail breakdown of AMC costing model estimates under different scenarios. The costing model was broken down into seven cost inputs: personnel (salaries and training), surgical equipment and supplies, bandages and cleaning supplies, monitoring and evaluation (M & E), administration, rural coverage, and adverse events due to the surgery. Fieno et al 2008 [41] assumed the value of medical care per HIV infection was to be the cost of two years of anti-retroviral therapy (ART) (US$500) in Mozambique or US$1000. Bollinger et al 2009 [43] used unit cost of an uncomplicated AMC of US$48 in the public sector as provided by Botswana national strategy. In addition, Bollinger et al 2009 [43] also developed three other unit costs: (1) a neonatal circumcision unit cost of US$38 (assumed to be 20% lower than the adult cost, due to lower complication rates and lower costs for commodities), (2) private provider unit costs for both adult and neonatal circumcisions of US$60 and US$48 (assumed to be 5% higher than the relevant public sector costs)., and (3) user fees of US$1 for public sector and US$25 for private providers were assumed.

Kahn et al. 2006 [39] defined effectiveness as the number of HIV infections prevented per 1,000 newly circumcised men over a specified number of years. The effectiveness was calculated as the product of the number of HIV susceptible, the HIV incidence rate, the protective effect of AMC (adjusted for risk compensation), the projection period (in years), and an epidemic multiplier. Kahn et al. 2006 [39] estimated net DALYs by subtracting the increase in DALYs due to adverse events from the reduction in DALYs due to HIA. The reduction in DALYs from HIV was calculated by multiplying HIA by previously reported discounted DALY changes with ART (ten DALYs) and without (21 DALYs), assuming 50% on ART. Gray et al 2007 [40] calculated the number of HIV infections potentially averted by each surgery from the total number of incident cases expected in the population in the absence of a circumcision program minus the number of incident cases estimated with varying circumcision efficacies. Auvert et al 2008 [41] estimated effects of AMC on HIV as a function of time and parameters were based on published studies. Fieno et al 2008 [41] assumed the number of DALYs per HIV/AIDS mortality to be 15.5 years. The benefits of AMC were estimated from the Kahn et al. 2006 [39] cost-effectiveness model. Bollinger et al 2009 [43] calculated number of AMC that are required in order to avert one HIV infection by dividing the increase in the number of AMC performed by the number of HIA over the relevant time period.

#### 7. Were costs and consequences adjusted for differential timing?

Most studies [39], [41], [42], [43] discounted cost accrued beyond 1 year. Kahn et al 2006 [39] was the only study that reported that future benefit was discounted.

#### 8. Was an incremental analysis of costs and consequences of alternatives performed?

All studies [39], [40], [41], [42], [43] calculated program cost per HIA. Fieno et al 2008 [42] also calculated the cost of one DALY saved from HIV infection averted.

#### 9. Was allowance made for uncertainty in the estimates of costs and consequences?

Except for Fieno et al 2008 [42], studies reported at least one form of sensitivity analyses. Gray et al 2007 [40] explored the effect of variation program coverage and circumcision efficacy on the cost per HIA. Kahn et al 2006 [39] conducted sensitivity analyses to examine the effects of all input uncertainty and program coverage using one-way, three-way and multivariate (using Monte Carlo simulations) sensitivity analyses. Auvert et al 2008 [41] explored a public cost scenario assuming the use of government health infrastructures only and a private cost scenario assuming reliance on the private health care providers only. In addition, Auvert et al 2008 [41] also considered the effect of different time horizon and program coverage on cost per HIA. Bollinger et al 2009 [43] examined the effects in variation in program effectiveness, discount rate, and lifetime cost of ART on the cost per HIA.

#### 10. Did the presentation and discussion of study results include all issues of concern to users?

Most of the studies discussed issue of availability, affordability, and sustainability of their findings.

### Outcome Measures

Table 1 also shows the estimated cost per HIA from the five studies. Gray et al 2007 [40] reported that the cost per HIA over 10 years could range from US$2808 with 60% circumcision efficacy, to US$4173 with 40% circumcision efficacy. Kahn et al. 2006 [39] estimated the cost per HIA over 20 years at US$193. Kahn et al. 2006 [39] also reported that the cost-effectiveness model was sensitive to the cost of AMC, cost of averted HIV treatment, the protective effect of AMC, and HIV prevalence. With an HIV prevalence of 8.4%, the cost of per HIA increased nearly 3-fold to US$588. Cost-effectiveness improves by less than US10$ when AMC intervention coverage is 50% of full coverage. Auvert et al 2008 [41] found that the estimated costs per HIA over 10 and 20 years were US$351 (US$271 to US$473) and US$174 (US$138 to US$232) respectively. Fieno et al 2008 [41] estimated the cost per HIA over 20 years at US$390. Bollinger et al 2009 [43] estimated the cost per HIA with 60% circumcision efficacy at US$642. However, the cost-effectiveness model was sensitive to AMC effectiveness. At 75% and 30% circumcision efficacy, the costs per HIA were US$508 and US$1313 respectively.

The reported cost savings varied across the studies. Kahn et al. 2006 [39] found that after adjustment for averted lifetime HIV medical costs, the net saving was US$2573 per AMC. Auvert et al 2008 [41] found that the estimated net savings over 20 years were US$2.4 billion (95% percentile interval 1.5 to 3.5). Fieno et al 2008 [41] reported that the saving from the AMC in terms of medical costs alone would accrue over 20 years to US$234.0 million. Bollinger et al 2009 [43] estimated net savings over 17 years with 60% circumcision efficacy at US$10,616. Only study reported cost per DALYs averted [41]. Fieno et al 2008 [41] reported that cost was US$7.8 per DALY.

## Discussion

### Main Findings

This systematic review of economic evaluations of adult male circumcision (AMC) for prevention of heterosexual acquisition of HIV in men provides compelling evidence that AMC is within the range of what is generally regarded as cost-effective, even cost-saving, intervention. The reported cost per HIA ranged from US$174 in Auvert et al 2008 [41] to as much as US$2808 in Gray et al 2007 [40]. The key driver of cost-effectiveness models was circumcision efficacy. Except for Gray et al 2007 [40], studies used evidence of effectiveness from OF trial [7]. Thus, possible explanation for the high cost per HIA reported by Gray et al 2007 [40] in comparison to other studies could be due to the fact that the model effectiveness data was based on observational study. However, these findings are comparable with other prevention and treatment strategies in developing countries in terms of economic criteria. Recent reviews of HIV prevention cost-effectiveness suggest a range of $10 to more than $10,000 per HIA [46], [47], [48]. Commercial sex worker interventions and mass-media campaign cost just over US$50 per HIA. Second-generation female condom cost US$985 per HIA. Blood safety measures cost US$41–246 per HIA. Diagnosis and treatment of sexually transmitted infections cost US$250 per HIA. Voluntary testing and counselling cost S$264–367 per HIA; and intervention for reducing mother-to-child transmission cost US$84–714 per HIA.

### Study Strengths and Limitations

The strengths of this study are that, to our knowledge, it provides the most comprehensive review to date of economic evaluations of male circumcision for prevention of heterosexual acquisition of HIV in men in sub-Saharan; and it is the first review in this area to critique the quality of modelling approaches used in the economic evaluations. The search process was elaborate and to our knowledge no other studies were available for review. In addition, we used a standard checklist to appraise the quality of economic evaluations. The reporting of this review conforms to PRISMA guideline [27], [28].

There are number of limitations associated with these studies. Most of these studies did not considered complications associated with AMC in their cost-effectiveness models. It has been reported that high complication rates challenge the implementation of male circumcision for HIV prevention in Africa [49]. Another important limitation of these studies, except for Kahn et al 2006 [39], most of the authors did not considered multivariate sensitivity analysis. The uncertainty in the evidence base needs to be reflected in the model. To simultaneously assess the implications of uncertainty in all elements of evidence, probabilistic analysis should be used to establish the decision uncertainty associated with each public health intervention being compared [50], [51]. This informs decision-makers about the probability of each strategy being the most cost-effective conditional on the value that the decision maker places on a unit of health gain. Such methods can also be used to provide an opportunity to apply value of information (VOI) methods to inform priority setting in research [52], [53], [54]. Generalizability of the findings is also an important limitation. Most of the studies were based on OF trial [7]. The OF trial [7] was conducted in a single country and used prevailing or local prices to calculate costs. Economic evaluation carried out alongside a randomised controlled trial may differ significantly from usual practice or care [55]. We recommend that future economic evaluations address these limitations and be guided in part by the checklists available for assessing economic evaluations. Economic evaluation provides a useful framework to assist policy makers in allocating resources across competing needs. HIV/AIDS is a considerable burden on society resources, and prevention provides a cost-beneficial solution to address these consequences. To better inform the decision-making process, researchers must continue to produce high-quality, methodological, comparable and scientifically credible economic evaluations.

All published economic evaluations offered the same conclusion that AMC is cost-effective and potentially cost-saving for prevention of heterosexual acquisition of HIV in men. On these grounds, AMC may be seen as a promising new form of strategy for prevention of HIV. However, we believed like others that AMC should never replace other known methods of HIV prevention. AMC should be considered as part of a comprehensive HIV prevention package, such as, promoting delay in the onset of sexual relations, abstinence from penetrative sex and reduction in the number of sexual partners; providing and promoting the correct and consistent use of male and female condoms; providing HIV testing and counselling services; and providing services for the treatment of sexual transmitted infections.
